# Transforming Growth Factor-β1 and Receptor for Advanced Glycation End Products Gene Expression and Protein Levels in Adolescents with Type 1 Diabetes Mellitus

**DOI:** 10.4274/jcrpe.galenos.2020.2020.0155

**Published:** 2021-02-26

**Authors:** Ana Ninić, Dragana Bojanin, Miron Sopić, Marija Mihajlović, Jelena Munjas, Tatjana Milenković, Aleksandra Stefanović, Jelena Vekić, Vesna Spasojević-Kalimanovska

**Affiliations:** 1 University of Belgrade Faculty of Pharmacy, Department for Medical Biochemistry, Belgrade, Serbia; 2 Mother and Child Health Care Institute of Serbia “Dr Vukan Čupić”, Biochemical Laboratory, Belgrade, Serbia; 3 Mother and Child Health Care Institute of Serbia “Dr Vukan Čupić”, Department of Endocrinology, Belgrade, Serbia

**Keywords:** Transforming growth factor-β1, receptor for advanced glycation end products, type 1 diabetes, urinary albumin excretion rate, quantitative polymerase chain reaction

## Abstract

**Objective:**

Type 1 diabetes (T1D) mellitus is one of the most frequent autoimmune diseases in childhood. Chronic complications are the main causes of cardiovascular morbidity and mortality in T1D. Although interactions between advanced glycation end products (AGE) and their receptors (RAGE) and transforming growth factor-β1 (TGF-β1) are implicated in development and progression of diabetic microand macro-vascular complications, they also have important roles in immune system regulation.

**Methods:**

Blood samples were obtained from 156 adolescents with T1D and 80 apparently healthy controls. T1D patients diagnosed with any other autoimmune disease and receiving any kind of drugs except insulin therapy were excluded from this study. Exclusion criteria for controls were positive family history of T1D and drugs/supplements application. TGF-β1 and transmembrane full-length RAGE (flRAGE) messenger ribonucleic acid (mRNA) levels in peripheral blood mononuclear cells (PBMC) were obtained by quantitative polymerase chain reaction (qPCR) method. Circulating levels of biochemical markers, TGF-β1 and soluble RAGE (sRAGE) levels were also determined.

**Results:**

TGF-β1 and flRAGE mRNA levels were significantly higher in controls compared to patients (p<0.001, for both). However, TGF-β1 and sRAGE levels were higher in patients than controls (p<0.001, for both). There were significant independent associations of all mRNA and protein levels with T1D. TGF-β1 mRNA was the only marker independently negatively associated with urinary albumin excretion rate in T1D adolescents (p=0.005).

**Conclusion:**

Our results indicated gene expression downregulation of TGF-β1 and flRAGE in PBMC of T1D adolescents. TGF-β1 mRNA downregulation may be useful for predicting early elevation of urinary albumin excretion rate.

**What is already known on this topic?**

As the non-enzymatic glycation products rise under conditions of chronic hyperglycemia, advanced glycation end products (AGE) through interaction with its receptor (RAGE) activates a range of signaling pathways which play an important role in the pathogenesis of diabetic complications (nephropathy, retinopathy, neuropathy and atherosclerosis). Transforming growth factor-β1 (TGF-β1) as a multifunctional cytokine, exerts pleiotropic effects from differentiation and development to cell growth and immunity regulation. Induced by many factors, including hyperglycemia, TGF-β1 is an important mediator in the pathogenesis of diabetic nephropathy, mainly stimulating the production of extracellular matrix components.

**What this study adds?**

The decrease in TGF-β1 gene expression in peripheral blood mononuclear cells significantly independently correlated with type 1 diabetes (T1D) and might be used as a potential biomarker for early cardiovascular risk assessment in adolescents with T1D by predicting early elevation of urinary albumin excretion rate. Decrease in transmembrane full-length RAGE gene expression, increase in TGF-β1 and soluble sRAGE concentrations could serve as biomarkers independently associated only with T1D presence.

## Introduction

Type 1 diabetes (T1D) mellitus is a chronic, T lymphocyte-mediated autoimmune disease leading to destruction of pancreatic Langerhans β-cells, endogenous insulin secretion decline and consequent hyperglycemia (1). Although, autoantibodies for β-islet cell constituents are mediate the ongoing autoimmune process and are useful in T1D diagnosis (2), other markers that reflect T lymphocyte activities can be used (3). Growing knowledge about gene expression changes in regulatory and effector immune cells, as new biomarkers, could provide novel insights into the pathogenesis of T1D (3,4). Autoreactive T cells, together with immune cells such as monocytes and other lymphocytes, which target islet β-cells, are capable of inducing T1D development.

Chronic hyperglycemia, either good or poorly regulated, causes the formation of advanced glycation end products (AGEs), which are a large group of irreversibly modified proteins (5). Contrary to early glycated end products (Schiff bases and Amadori products such as fructosamine) (5), AGEs express their effects by binding to transmembrane, full-length receptor for AGEs (flRAGE) (6). flRAGE on interacting with AGE elicits a loss of balance in oxidant and antioxidant production and gives rise to inflammatory and thrombogenic responses in endothelial cells (7). Accordingly, AGE-flRAGE interaction has been reported to induce vascular stiffening, angiogenesis, and extracellular matrix (ECM) accumulation (5,8). Through these mechanisms, flRAGE-AGE interaction exerts major effects in chronic micro- and macro-vascular complications (nephropathy, neuropathy, retinopathy, and atherosclerosis) development. Also, enhanced flRAGE expression interacting with AGE in T cells may induce their inflammatory functions, leading to β-cell injury during T1D progression (9). However, flRAGE activation may also suppress autoimmune responses by activation of regulatory T cells (Treg) (10).

Circulating RAGE isoforms, termed soluble RAGE (sRAGE), can bind AGEs and thus prevent their detrimental effects following flRAGE activation (11). In this manner, sRAGE exhibits a protective role. It has been reported that by blocking flRAGE, sRAGE exerted an anti-atherogenic effect through inhibiting cell migration and enlargement of atherosclerotic lesions (12). However, other studies demonstrated that sRAGE induced vascular permeability by increasing production of proinflammatory and chemo-attractant molecules, thus maintaining vascular inflammation and progression of atherosclerotic process (13).

As multifunctional cytokine, transforming growth factor-β1 (TGF-β1) stimulates production of ECM proteins including collagen 1 and 4, laminin, and fibronectin, thus participating in extracellular remodelling in peripheral organs leading to development of micro- and macro-vascular chronic complications (14,15). TGF-β1 is likely to be an essential factor in the development of diabetic nephropathy. On the other hand, TGF-β1 secreted by Tregs has a role in autoreactive T cells suppression, induction of immune tolerance and inhibition of proinflammatory cytokine production (14). These opposing effects of TGF-β1 make it an interesting biomarker for early evaluation of T1D and its chronic complications development.

The association of AGE-flRAGE and TGF-β1 exists during development of diabetes and diabetic nephropathy. TGF-β-activated kinase 1 stimulated by AGEs, plays an important role in innate immune responses and inflammation, activating main proinflammatory pathways (mitogen-activated protein kinases - MAPKs and nuclear factor-κB), macrophage polarization from M2 to M1, and inflammatory cytokine production (16,17). Moreover, AGEs stimulate fibrotic processes leading to the appearance of myofibroblasts and the accumulation of ECM components via the TGF-β1-independent Smad3 signaling pathway (18).

Accordingly, the first aim of this study was to investigate whether changes in TGF-β1 and flRAGE gene expression in peripheral blood mononuclear cells (PBMCs) and TGF-β1 and sRAGE serum protein levels were associated with the presence of T1D autoimmunity. Our second goal was to contribute to current knowledge by identifying whether the expression of these genes and protein levels could be related to suboptimal/poor glycemic control and future diabetic chronic complications, such as early elevation of urinary albumin excretion rate.

## Methods

Adolescents with T1D and apparently healthy adolescents as controls were enrolled in the study. The groups were matched by age, pubertal stage, and body mass index (BMI). The participants were recruited from the Mother and Child Health Care Institute of Serbia “Dr. Vukan Čupić”, Belgrade, during regular follow in the outpatient clinic. Diabetes was diagnosed according to Serbian national and international guidelines of good clinical practice for diagnosis and treatment of diabetes mellitus (19,20). Patients with T1D were treated with intensive insulin therapy given as a basal-bolus regimen. Good, suboptimal/poor glycemic control in T1D patients was defined according to a glycated hemoglobin A1c (HbA1c) concentration cut-off of 7.5% (21). Exclusion criteria for T1D patients were: medicaments usage (except insulin); presence of cystic fibrosis; presence of other autoimmune diseases such as thyroiditis and celiac disease. Exclusion criteria for the control group were positive family history of T1D and use of drugs and supplements.

In all study participants the following evaluations were performed: demographic data collection, clinical data collection including age, body weight, body height, BMI, puberty staging, age at diabetes onset, diabetes duration, insulin dosage and laboratory tests included blood glucose, creatinine, HbA1c, and C-reactive protein (CRP), urinary albumin and glomerular filtration rate (GFR), and TGF-β1 and RAGE gene expression and protein levels.

Body weight was measured to the nearest 0.1 kg by a portable electronic scale (Tanita, Amsterdam, Netherlands). Body height was measured to the nearest 0.1 cm using a portable wall-mounted stadiometer. BMI was calculated as body weight (kilograms) divided by the squared height (meters). Puberty stages were stratified by clinical examination according to Tanner (22).

Blood samples from all study participants were collected into two serum and one ethylenediaminetetraacetic acid containing vacutainers (BD Vacutainer®, New Jersey, USA). After venepuncture, serum and plasma were immediately separated and stored at -80 °C prior analyses.

Glucose and creatinine were assayed in serum samples using routine laboratory methods. HbA1c level was determined by competitive turbidimetric inhibition immunoassay. CRP was measured using immunoturbidimetric method. All the analyses were performed on Roche/Hitachi c501 automated analyser (Roche, Mannheim, Germany). Urinary albumin was determined in the timed overnight sample using a nephelometer, the Siemens BN ProSpec® System (Siemens, Erlangen, Germany). Early elevation of albumin excretion rate cut-off was set as ≥7.5 µg/min because these values were shown to predict subsequent persistent microalbuminuria development later in life (23). Estimated glomerular filtration rate (eGFR) was assessed using the Schwartz equation. The TGF-β1 protein levels were determined in serum samples and sRAGE protein levels were measured in plasma, using enzyme-linked immunosorbent assays according to the manufacturer’s manual (DuoSet, R&D systems, Wiesbaden, Germany).

PBMCs were isolated after plasma separation using the Ficoll-Paque® PLUS gradient-gel (GE Healthcare, Wisconsin, USA) according to the manufacturer’s instructions. After isolation, but before freezing at -80 °C, PBMC were suspended in 1 mL of TRIzol^TM^ reagent (Invitrogen Life Technologies, Foster City, USA).

Total ribonucleic acid (RNA) from PBMCs was isolated using a modified classic RNA isolation protocol, described by Chomczynski (24), which was optimized for the laboratory of the Department for Medical Biochemistry, University of Belgrade-Faculty of Pharmacy, explained in detail and published elsewhere (25).

Reverse transcription and quantitative real-time polymerase chain reaction (qPCR) experiments were performed on the 7500 real-time PCR System using Assays-on-Demand based on TaqManTM chemistry (Applied Biosystems, Foster City, USA).

The qPCR reactions were performed using TaqMan^TM ^5’-nuclease gene expression assays (Applied Biosystems, Foster City, USA) for TGF-β1 (Hs00998133_m1) and flRAGE (Hs00153957_m1) genes. The relative standard curve method was used for gene expression quantification. Relative gene expression levels were expressed as a ratio between target gene and constitutively expressed gene as housekeeping gene (β-actin) messenger RNA (mRNA) using the following equations:

Normalised TGF-β1 mRNA levels = TGF-β1 mRNA/β-actin mRNA

Normalised flRAGE mRNA levels = flRAGE mRNA/β-actin mRNA.

Negative controls for reverse transcription (no reverse transcriptase enzyme) and for qPCR (no complementary deoxyribonucleic acid) were included in the experiments.

The study was carried out in line with the principles of the Declaration of Helsinki and approved by the Ethics Committees of Mother and Child Health Care Institute of Serbia “Dr. Vukan Čupić” (protocol number: 8/8, date: April the 9^th^ 2015) and University of Belgrade-Faculty of Pharmacy (protocol number: 2536/2, date: December 26^th^ 2018). Written informed consent was obtained from all the participants and their parents.

### Statistical Analysis

All statistical testing was performed using a statistical program, SPSS Statistics, version 22 (IBM Inc., Chicago, IL., USA). Distribution of continuous variables was tested by Kolmogorov-Smirnov test. Continuous normally distributed data are presented as arithmetic mean±standard deviation. If normal distribution was not achieved after logarithmic transformation, skewed distributed data were presented as median (interquartile range).

Comparisons between the tested groups were made using Student’s *t*-test for normally distributed data and by Mann-Whitney *U* and Kruskal-Wallis tests for skewed distributed data. Categorical data were given as absolute frequencies and compared by chi-square test for contingency tables. Associations between clinical data were tested by Spearman’s bivariate correlation analysis. In-depth possible associations of tested markers with T1D and urinary albumin excretion rate were assessed by univariate and multivariate binary logistic and ordinal regression analyses, respectively. Binary logistic regression analysis was used to find single predictors and models (mRNA and protein levels) as explanatory variables associated with T1D presence. Ordinal regression analysis was used as a statistical test to determine potential associations of single markers and models and T1D complication (early elevation of urinary albumin excretion rate). In multivariate ordinal regression analysis, there were no multicollinearity between independent variables (predictors) and all of them had an identical effect at each cumulative split of the ordinal dependent variable (urinary albumin excretion rate quartiles). Data from bivariate correlation are presented as correlation coefficient (ρ). Data from binary logistic and ordinal regression analyses are presented as odds ratio (OR) and 95% confidence interval (CI). Explained variations in T1D and urinary albumin excretion rate were assessed by Nagelkerke R^2^. The statistically significant level was set at p<0.05.

## Results

### Clinical and Laboratory Data in Tested Groups

156 adolescents with T1D and 80 controls were enrolled in the study. The median age of the T1D adolescents (49% females) was (12,13,14,15,16) years. The median T1D duration was 7 (5,6,7,8) years. In the T1D group, 33 (21%) had good glycemic control and 123 (79%) had suboptimal/poor glycemic control. In the control group of 80 participants (82% females) median age was 15 (13,14,15,16,17) years. General anthropometric and biochemical data of tested populations were presented in the Table 1. No statistically significant differences between T1D patients and the controls were found in terms of age and BMI. However, significantly more females than males were in the control group compared to the patient group (p<0.001). Adolescents in the control group had significantly lower glucose, HbA1c and CRP than patients with T1D (Table 1).

**Figure 1:**
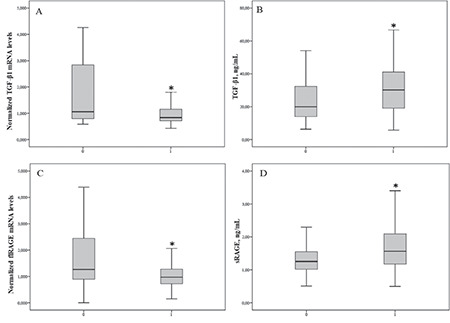
flRAGE and TGF-β1 normalised mRNA levels and protein concentrations between tested groups Data are presented as median (interquartile range) and compared by Mann-Whitney U test. *p<0.001. 0-Control group; 1- T1D. T1D: type 1 diabetes mellitus, TGF-β1: transforming growth factor-β1, flRAGE: full-length receptor for advanced glycation end product, sRAGE: soluble receptor for advanced glycation end products, mRNA: messenger ribonucleic acid

Patients with T1D had significantly lower TGF-β1 mRNA and flRAGE mRNA levels (Figure 1A, 1C), but higher TGF-β1 and sRAGE protein concentrations than controls (Figure 1B, 1D).

In order to examine whether discrepant gender distribution in tested groups could affect obtained results, we first compared clinical markers between males and females of the control group. Then we compared only females and finally we compared only males between patient and control groups. There were no significant differences in any clinical marker between genders (66 females vs 14 males) of the control group. The same trends for examined markers were found between females from control group (n=66) vs patient group (n=76) and males from control group (n=14) vs patient group (n=80) as we already obtained when we analysed both genders joined in each examined group (Tables 1, 2, Figure 1). Briefly, glucose, HbA1c, CRP, TGF-β1, and sRAGE levels were significantly higher in patients compared to controls for each gender. TGF-β1 mRNA and flRAGE mRNAs were significantly lower in patients compared to controls for each gender. Accordingly, we performed further statistical analysis in the initially formed groups.

### Binary Logistic Regression of mRNA and Protein Levels for Association with T1D

We further investigated whether TGF-β1 mRNA and flRAGE mRNA levels and TGF-β1, sRAGE and CRP levels were associated with T1D (Table 2). Significant ORs obtained in univariate binary logistic regression analysis were evident for all tested markers, indicating their significant associations with T1D. Nagelkerke R^2^ showed that each predictor TGF-β1 mRNA, flRAGE mRNA, sRAGE protein, TGF-β1 protein and CRP, in univariate analysis could explain the variation in T1D development by 24.1%, 17.8%, 14%, 8.2% and 6.6%, respectively. These predictors were further adjusted for demographic and laboratory variables (gender, age and CRP), which were significantly different between tested groups or implicated in T1D development, to assess their possible independent associations with T1D. As presented by the Model 1 TGF-β1 mRNA and flRAGE mRNA levels were independently negatively associated with T1D (OR=0.284, p<0.001 and OR=0.396, p<0.001, respectively). On the other hand, CRP, TGF-β1 and sRAGE protein levels was independently positively associated with T1D (OR=1.438, p=0.018, OR=1.037, p=0.002 and OR=3.552, p<0.001, respectively) (Table 2).

### Correlation Analyses of mRNA and Protein Levels with Other Clinical and Laboratory Markers in T1D Patients

Next, we conducted Spearman’s correlation analysis to test bivariate associations between TGF-β1 mRNA, flRAGE mRNA, TGF-β1 and sRAGE protein levels with other markers in patients with T1D (Table 3). It was found that lower TGF-β1 mRNA levels were associated with older age of diabetes onset and higher urinary albumin excretion rate. Lower flRAGE mRNA levels were related to higher serum creatinine levels and lower eGFR. sRAGE and TGF-β1 protein levels correlated positively with diabetes duration and negatively with eGFR. Also, TGF-β1 protein concentration correlated positively with age and creatinine level (Table 3). Furthermore, TGF-β1 mRNA and flRAGE mRNA levels were in mutual positive correlation, as were sRAGE and TGF-β1 protein concentrations. There were no significant correlations either between TGF-β1 mRNA and its protein levels or between flRAGE mRNA and sRAGE levels.

### mRNA and Protein Levels According to Glycemic Control in T1D Patients

Additionally, we wanted to test whether good or suboptimal/poor metabolic control in T1D could influence TGF-β1 mRNA and flRAGE mRNA, and TGF-β1 and sRAGE protein levels, as well as CRP concentration. Consequently, we divided the T1D group into two subgroups (0 - HbA1c <7.5%; 1 - HbA1c ≥7.5%). No significant differences in mRNA and protein levels, according to glycemic control were identified. Also, no significant correlations were evident between HbA1c and mRNA and protein levels in T1D patients (Table 3). However, when comparing CRP concentrations between the groups, adolescents with suboptimal/poor glycemic control (median: 0.85 mg/L, interquartile range: 0.40-2.30 mg/L) had significantly higher CRP concentration (p=0.020) than those with good glycemic control (median: 0.50 mg/L, interquartile range: 0.30-0.75 mg/L).

### mRNA and Protein Levels According to Urinary Albumin Excretion Rate Quartiles in T1D Patients

**Figure 2:**
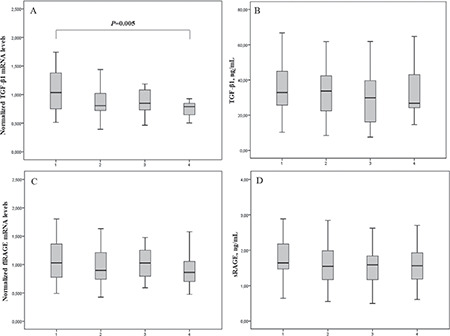
TGF-β1 and flRAGE mRNA levels, TGF-β1 and sRAGE protein concentration in adolescents with type 1 diabetes mellitus according to urinary albumin excretion rate quartiles Data are presented as median (interquartile range) and compared by Kruskal-Wallis and Mann-Whitney U tests. 1) the first quartile (≤2.92 μg/min); 2) the second quartile group (2.93- 4.24 μg/min); 3) the third quartile group (4.25-7.49 μg/min); 4) the fourth quartile group (≥7.5 μg/min). Each quartile consisted of 39 participants. TGF-β1: transforming growth factor-β1, flRAGE: full-length receptor for advanced glycation end product, sRAGE: soluble receptor for advanced glycation end products, mRNA: messenger ribonucleic acid

Our further intention was to determine whether TGF-β1 mRNA and flRAGE mRNA and TGF-β1 and sRAGE protein levels were associated with early elevation of urinary albumin excretion rate in T1D patients. To achieve this, urinary albumin excretion rate values were divided into quartiles. Each quartile consisted of 39 participants. Early elevation of albumin excretion defined as ≥7.5 µg/min corresponds to the fourth quartile. TGF-β1 mRNA levels were significantly different between quartiles (p=0.025) being lower in the fourth than in the first quartile group (p=0.005) (Figure 2A). There were no significant differences in other tested markers between urinary albumin excretion rate quartile groups (Figure 2B, 2C, 2D). Also, we were not able to determine significant differences in CRP concentration between quartile groups (p=0.967) (data not presented in Figure 2).

### Ordinal Regression Analysis of TGF-β1 mRNA Levels for Association of Early Elevation of Urinary Albumin Excretion Rate in T1D Patients

Due to the significantly high negative correlation between TGF-β1 mRNA and urinary albumin excretion rate in T1D patients (Table 3), our further intention was to determine whether an in-depth association between them existed. To achieve this, univariate and multivariate ordinal regression analysis was performed. TGF-β1 mRNA levels showed significant ORs for urinary albumin excretion rate in univariate analysis (OR=0.278, 95% CI: 0.126-0.612, p=0.001). Nagelkerke R^2^ for TGF-β1 mRNA levels was 0.140. Multivariate analysis revealed an independent association of TGF-β1 mRNA levels with early elevation of urinary albumin excretion rate when tested with other clinical variables which might be implicated in its elevation. Those variables were age, diabetes duration, CRP and HbA1c. A decrease in TGF-β1 mRNA levels increased the probability of elevation of urinary albumin excretion rate (OR=0.309, 95% CI: 0.136-0.698, p=0.005). Nagelkerke R^2^ of 0.162 indicated that multivariate regression model could explain 16.2% variation in urinary albumin excretion rate.

## Discussion

The present study demonstrated that a decrease in TGF-β1 mRNA levels was independently associated with T1D and early elevation of urinary albumin excretion rate, while CRP, TGF-β1 and sRAGE protein concentrations were independently associated with T1D only. In addition, adolescents with T1D expressed lower flRAGE mRNA levels than controls, which were independently associated with T1D, but not to early elevation of urinary albumin excretion rate. None of the tested markers were related to glycemic control in T1D adolescents.

As an autoimmune disease accompanied by chronic inflammation, T1D tends to develop in childhood (1). Regardless, vascular complications can be detected in adolescents after five-years duration of T1D, indicating faster development than in adults (26). Such complications are one of the most important causes of mortality in T1D patients. Although assessment of autoantibodies in blood is the gold standard for identification of patients with T1D or at-risk patients (27), new biomarkers are required to indicate and assess β-cells destruction and monitor T1D progression in clinical practice (4).

It is very difficult to obtain samples of body organs, e.g. pancreas, blood vessel endothelium or kidneys in adolescents. However, PBMC, lymphocytes and monocytes, can serve as surrogate cells for RNA isolation and gene expression determination (28). Gene expression profiles in peripheral blood immune cells may provide new insights into the T1D pathogenesis (4,28). Still, there are contradictory results and conflicting explanations concerning RAGE and TGF-β1 gene expression in PBMC in T1D patients published to date (3,29,30,31,32,33).

TGF-β1, as multifunctional cytokine, is produced by virtually all cells in humans, and has contradictory effects depending on the tissue being assessed (14,15). Demonstrated by Saxena et al (14), TGF-β1 plays a dual role during the development and progression of systemic autoimmune-inflammatory disease in mice. Reduced TGF-β1 synthesis by immune cells indicated autoimmune onset in early life. Also, TGF-β1 produced by Treg cells was shown to inhibit autoantibody synthesis. On the other hand, increased TGF-β1 synthesis in other tissues predisposes local fibrogenesis and likely leads to organ damage, for example kidney injury (14). Results from our study supported these findings. We found significantly lower TGF-β1 gene expression in PBMC from T1D patients compared to healthy adolescents (p<0.001). On the other hand, TGF-β1 protein concentration in T1D patients was significantly higher than in controls (p<0.001). This was expected because TGF-β1 production is enhanced in other peripheral organs in children with T1D (34). Furthermore, although they were not in mutual correlation, downregulation in TGF-β1 gene and increase in TGF-β1 protein levels were found to be independently associated with T1D development (p<0.001 and p=0.002, respectively). Adolescents with lower TGF-β1 mRNA levels were 71.6% more likely to exhibit T1D than those with higher levels. However, the odds of having T1D was 1.037 times greater in adolescents with higher TGF-β1 concentration.

TGF-β1 downregulation has been previously reported in PBMC of children with T1D, indicating depressed immunity in patients with long-term T1D (29). Tolerance against self-antigens can be maintained through activation of Treg cells that produce TGF-β1 (14). However, in our adolescents with T1D downregulation of TGF-β1, reflected as lower mRNA levels than in controls was evident. This apparently suggested maturation of T helper 1 lymphocytes, which could have been implicated in destruction of pancreatic β-cells (35). Nevertheless, dysregulation of TGF-β1 expression by Treg cells occurs even during the pre-diabetic stage (29). Children positive for islet cell antibodies had significantly lower TGF-β1 mRNA levels than controls (29). In our study, another confirmation of immune cell dysregulation was demonstrated by flRAGE mRNA levels, which were lower in T1D patients than in controls (p<0.001). Membrane RAGE also modulates Treg cell function and suppresses the autoimmune response and the downregulation of membrane RAGE has been implicated T1D development (10,36). Decrease in flRAGE mRNA levels was found to be independently associated with T1D development (p<0.001). Adolescents with lower flRAGE mRNA levels were 60.4% more likely to exhibit T1D than those with higher levels. Additionally, positive correlation between TGF-β1 mRNA and flRAGE mRNA levels (p<0.01) was evident in the T1D, cohort suggesting potential interplay of their signalling pathways in T1D pathogenesis.

In contrast to our and other studies, Jin et al (33) published a study of gene expression profiles in human PBMC of T1D patients using microarray technology. They analysed 18 genes involved in inflammation and immunity, among which TGF-β1 showed higher expression in T1D patients than in controls. These authors suggested that increase in TGF-β1 gene expression may have a role in T1D through stimulation of proinflammatory cellular pathways in PBMC (33).

T1D as a chronic metabolic disease causes future micro- and macro-vascular complications (26). Disturbance in blood vessel integrity correlates with poor glycemic control and diabetes duration (37). Heier et al (37) demonstrated that after five years from diabetes onset, accelerated atherosclerosis was evident in children with T1D. Though microalbuminuria has been related to diabetic nephropathy development and progression (38), it has recently been recognized as an independent predictor for endothelial dysfunction and cardiovascular disease (CVD) (39).

In our T1D study participants, with an average diabetes duration of seven years, glycemic control as defined by HbA1c seemed not to have any influence on the main tested markers. TGF-β1 mRNA, flRAGE mRNA, TGF-β1 and sRAGE protein levels did not differ between T1D adolescents with good vs suboptimal/poor glycemic control. Also, there were no correlations between them and HbA1c. However, when urinary albumin excretion rate values were divided into quartiles, TGF-β1 mRNA levels were lower in the fourth compared to the first quartile group (p=0.005). The fourth quartile corresponds to early elevation of urinary albumin excretion rate that could predict permanent micro- and later macro-albuminuria (23). Interestingly, our results indicated independent associations of lower TGF-β1 mRNA levels with elevated urinary albumin excretion rate in T1D adolescents (p=0.005). Adolescents having T1D with lower TGF-β1 gene expression were 69.1% more likely to have elevated urinary albumin excretion rate than those with higher expression. In our group of T1D adolescents with average diabetes duration of seven years, TGF-β1 mRNA levels could be used as a potential biomarker for CVD risk assessment indicating not only dysregulation of immune response to autoantigens, but also predicting future cardiovascular complications. It was expected that TGF-β1 protein, a fibrogenic factor, would correlate with urinary albumin excretion rate (14). However, this was not supported by our results. Yet, TGF-β1 protein levels in blood correlated significantly positively with creatinine (p<0.05) and negatively with eGFR (p<0.05) linking TGF-β1 with potential future renal function decline in T1D patients.

Miura et al (30) demonstrated that lower cell surface RAGE expression in monocytes of children with T1D could be partly explained by enhanced ligand binding, indicating an imbalance in receptor function on monocytes making them more prone to modification in subcellular space. Moreover, patients with incipient or clinical diabetic nephropathy showed a significant decrease in monocyte RAGE mRNA levels compared to patients without nephropathy. Our results may support these findings. We did not determine AGE concentration in blood of our participants and were not able to explain lowering RAGE expression with AGE engagement as in the Miura et al (30) study, but a relationship between lowering flRAGE mRNA and future diabetic vascular complications was apparent in our study.

sRAGE, made by protein cleavage of the extracellular ligand binding domain of transmembrane RAGE, has been suggested to be a biomarker for vascular disease development (11,12,13). Our results indicated that higher sRAGE levels were independently associated with T1D (p<0.001). The OR of having T1D was 3.552 times greater in adolescents with higher sRAGE concentration. However, sRAGE was not related to urinary albumin excretion rate. It correlated significantly positively with diabetes duration (p<0.01) and negatively with eGFR (p<0.05). Not only mRNA levels, but TGF-β1 and sRAGE protein levels correlated significantly positively (p<0.01), pointing out to one more evidence for TGF-β1 and sRAGE proteins probable mutual implication in T1D pathogenesis. These results could support sRAGE potent pro-atherogenic effect in addition to its immunomodulating properties (11).

As T1D is an immuno-inflammatory disease, CRP levels were expected to be increased in adolescents with T1D (37). According to our results, CRP levels were significantly higher in patients with T1D than in the control group and were also independently associated with T1D. These results are in line with the fact that not only an autoimmune process, but inflammation could be related to destruction of islet β-cells (40).

Changes in TGF-β1 mRNA and flRAGE mRNA, as well as in TGF-β1 and sRAGE protein levels were evident between patients and controls. Disturbances in TGF-β1 and flRAGE gene expression levels in PBMC are detectable in the pre-diabetic stage and persist during development of T1D (10,29,36,41). Therefore, mRNA measurement would be beneficial to perform at an early age when there is a suspected onset of diabetes, for example in patients with a positive family history. The same can account for TGF-β1 and sRAGE protein levels. Also, TGF-β1 mRNA levels should be determined at T1D diagnosis for possible diabetes complications assessment (37,38). However, due to possible effects of acute and chronic hyperglycemia (5,41,42,43) on these markers, multiple prospective measurements would be strongly recommended.

### Study Limitations

This study has several limitations. Firstly, this research was carried out as a cross-sectional study, which demonstrated significant associations between tested markers and T1D presence and urinary albumin excretion rate, but was not able to assess causal relationships between them. However, our findings need to be confirmed in prospective studies to determine whether progressive downregulation of the TGF-β1 gene occurs as microalbuminuria worsens. Secondly, TGF-β1 and RAGE protein concentration determination in PBMC of patients and controls should be addressed in future studies, together with their mRNA levels, to demonstrate whether their protein levels were lower and correlated with lower mRNA levels in T1D and to confirm immunomodulatory dysfunction in those cells of T1D patients. Finally, although the presence of significantly more females than males in the control group did not skew the results and conclusions of this study, the inclusion of more males would have made our current findings more robust. Nevertheless, our current study might form the basis for future research.

## Conclusion

In conclusion, T1D onset progresses in tandem with lower PBMC TGF-β1 mRNA and flRAGE mRNA levels, together with increased secretion of the proinflammatory cytokines TGF-β1 and sRAGE by other cells, as well as systemic low-grade inflammation. In addition, downregulation of the TGF-β1 gene might be used as a potential biomarker for early CVD risk assessment in adolescents with T1D, due to its independent significant negative association with urinary albumin excretion rate.

## Figures and Tables

**Table 1:**
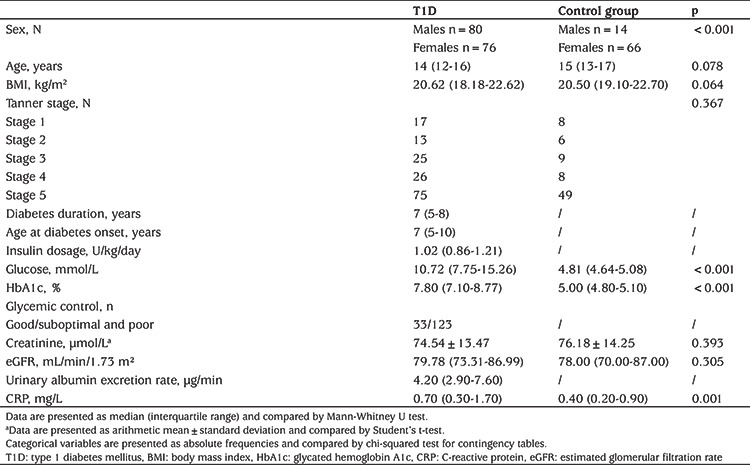
General characteristics and biochemical markers of two study groups

**Table 2:**
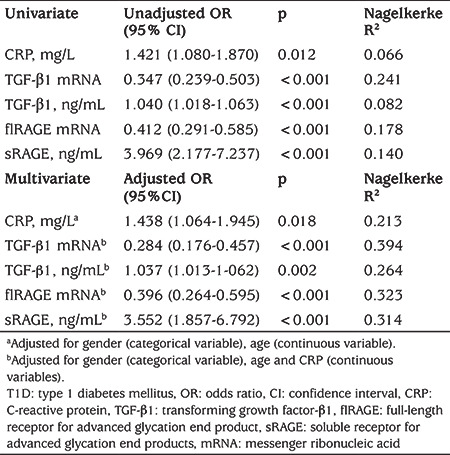
Univariate and multivariate binary logistic regression analysis for the associations of tested markers and type 1 diabetes mellitus development

**Table 3:**
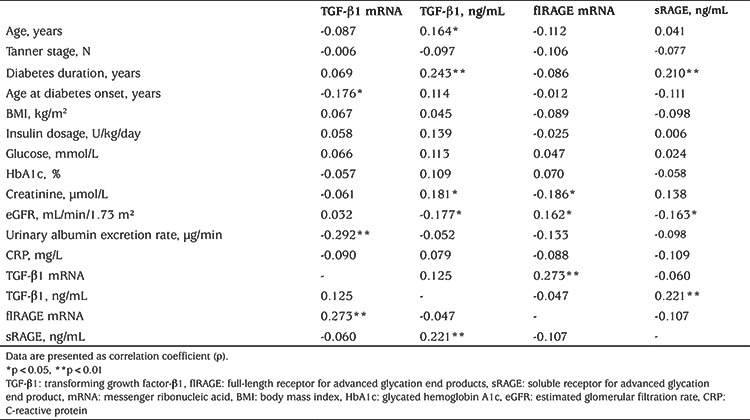
Significant correlations between flRAGE and transforming growth factor-β1 mRNA and protein concentrations with demographic data and biochemical markers in adolescents with type 1 diabetes mellitus
